# Predictors of disease activity in 857 patients with MS treated with interferon beta-1b

**DOI:** 10.1007/s00415-015-7862-9

**Published:** 2015-08-05

**Authors:** Hans-Peter Hartung, Ludwig Kappos, Douglas S. Goodin, Paul O’Connor, Massimo Filippi, Barry Arnason, Giancarlo Comi, Stuart Cook, Douglas Jeffery, John Petkau, Richard White, Timon Bogumil, Karola Beckmann, Brigitte Stemper, Gustavo Suarez, Rupert Sandbrink, Christoph Pohl

**Affiliations:** Department of Neurology, Heinrich Heine University, Düsseldorf, Germany; Neurology, University of Basel and University Hospital, Basel, Switzerland; Department of Neurology, University of California, San Francisco, CA USA; Division of Neurology, St. Michael’s Hospital, University of Toronto, Toronto, Canada; Neuroimaging Research Unit, Division of Neuroscience, Institute of Experimental Neurology, San Raffaele Scientific Institute, Vita-Salute San Raffaele University, Milan, Italy; Department of Neurology, University of Chicago Surgery Brain Research Institutes, Chicago, IL USA; Department of Neurology and Institute of Experimental Neurology, Università Vita-Salute San Raffaele, Milan, Italy; Department of Neurology and Neurosciences, Rutgers, The State University of New Jersey, Newark, NJ USA; Wake Forest University School of Medicine, Winston-Salem, NC USA; Department of Statistics, University of British Columbia, Vancouver, Canada; Bayer Pharma AG, Berlin, Germany; Department of Neurology, University Erlangen-Nürnberg, Erlangen, Germany; Bayer HealthCare Pharmaceuticals, Whippany, NJ USA; Department of Neurology, University Hospital of Bonn, Bonn, Germany

**Keywords:** Multiple sclerosis, Interferon beta-1b, MRI lesion, Relapse, Predictor

## Abstract

Multiple sclerosis (MS) is a chronic demyelinating neurodegenerative disease of the CNS that requires long-term treatment. The identification of patient characteristics that can help predict disease outcomes could improve care for patients with MS. The objective of this study is to identify predictors of disease activity in patients from the BEYOND trial. This regression analysis of patients with relapsing–remitting MS from BEYOND examined the predictive value of patient characteristics at baseline and after 1 year of treatment with interferon beta-1b 250 μg every other day for clinical and MRI outcomes after year 1 of the study. 857 and 765 patients were included in the analyses of clinical and MRI outcomes, respectively. In multivariate analyses of age, a higher number of relapses in the past 2 years, ≥3 new MRI lesions in the first year, and, especially, a higher number of relapses in year 1 predicted the future occurrence of relapses. By contrast, age, MRI activity, and the presence of neutralizing antibodies in the first year were principally predictive of future MRI activity. In patients with continued clinical disease activity or substantial MRI activity on therapy, an alternative therapeutic approach should be strongly considered.

## Introduction

MS is a chronic demyelinating disease of the CNS which often causes significant disability [1]. Many patients with MS will require treatment with disease-modifying therapies for the rest of their lives after diagnosis, and, therefore, the identification of predictors of disease course, both at the start of therapy and early in the course of treatment, might be helpful in planning the course of further therapy.

Interferon beta-1b (IFNβ-1b; Betaferon^®^/Betaseron^®^, Bayer HealthCare Pharmaceuticals, Whippany, New Jersey, United States) has been shown to be effective for the treatment of patients with relapsing–remitting MS (RRMS) in several clinical trials [2, 3]. Nevertheless, some patients may still have an unfavorable disease course despite treatment. Consequently, determining predictors of future disease course, especially those that take place during therapy with interferon beta-1b, may help guide management decisions [4]. To this end, we undertook a subgroup analysis of the BEYOND trial [3], which explored the value of using various clinical, MRI, and laboratory parameters as predictors of the future disease course in patients with RRMS treated with the standard dose of interferon beta-1b.

## Materials and methods

In the BEYOND study, patients were randomized to receive interferon beta-1b 250 µg or 500 µg sc every other day or glatiramer acetate 20 mg sc every day and were observed for a minimum of 2 years and for a maximum of 3.5 years. For the present analysis, only the data for patients who received the approved 250 µg dose of IFNβ-1b and who had been observed for ≥1 year were included. Clinical outcomes were assessed quarterly, and the presence of neutralizing antibodies (NAbs) was determined every 6 months using the MxA induction assay [5]. MRI assessment was done at screening and then annually thereafter. For a full description of the methods of the BEYOND study, see O’Connor et al. [3].

The prediction of post-year 1 disease course was evaluated using overdispersed Poisson regression models with relapse rate and MRI activity rate serving as the dependent variables. This method appropriately accounts for the different follow-up times for different patients. In univariate models, each predictor was examined separately. By contrast, for the multivariate models all predictors were incorporated simultaneously and a stepwise procedure (based on the Akaike information criterion) was used to select those with a significant and independent contribution to the model. The baseline predictors of age, EDSS score, number of relapses in the 2 years before study entry, disease duration, T2 volume, and T1 hypointensity (black hole) volume were analyzed as continuous variables. The baseline predictors of sex and the presence of any T1 gadolinium-enhancing (Gd+) lesions (yes or no) were analyzed as dichotomous variables. The categorical predictors based on year 1 disease activity were the number of new MRI lesions (0, 1–2, ≥3), NAb titer at year 1 (0, 20–400, ≥400 NU/mL), the occurrence of relapses (yes or no) within the first year of IFNβ-1b therapy, and the EDSS progression as assessed by a sustained change of ≥1.0 point, confirmed after 12 months.

## Results

**Fig. 1:**
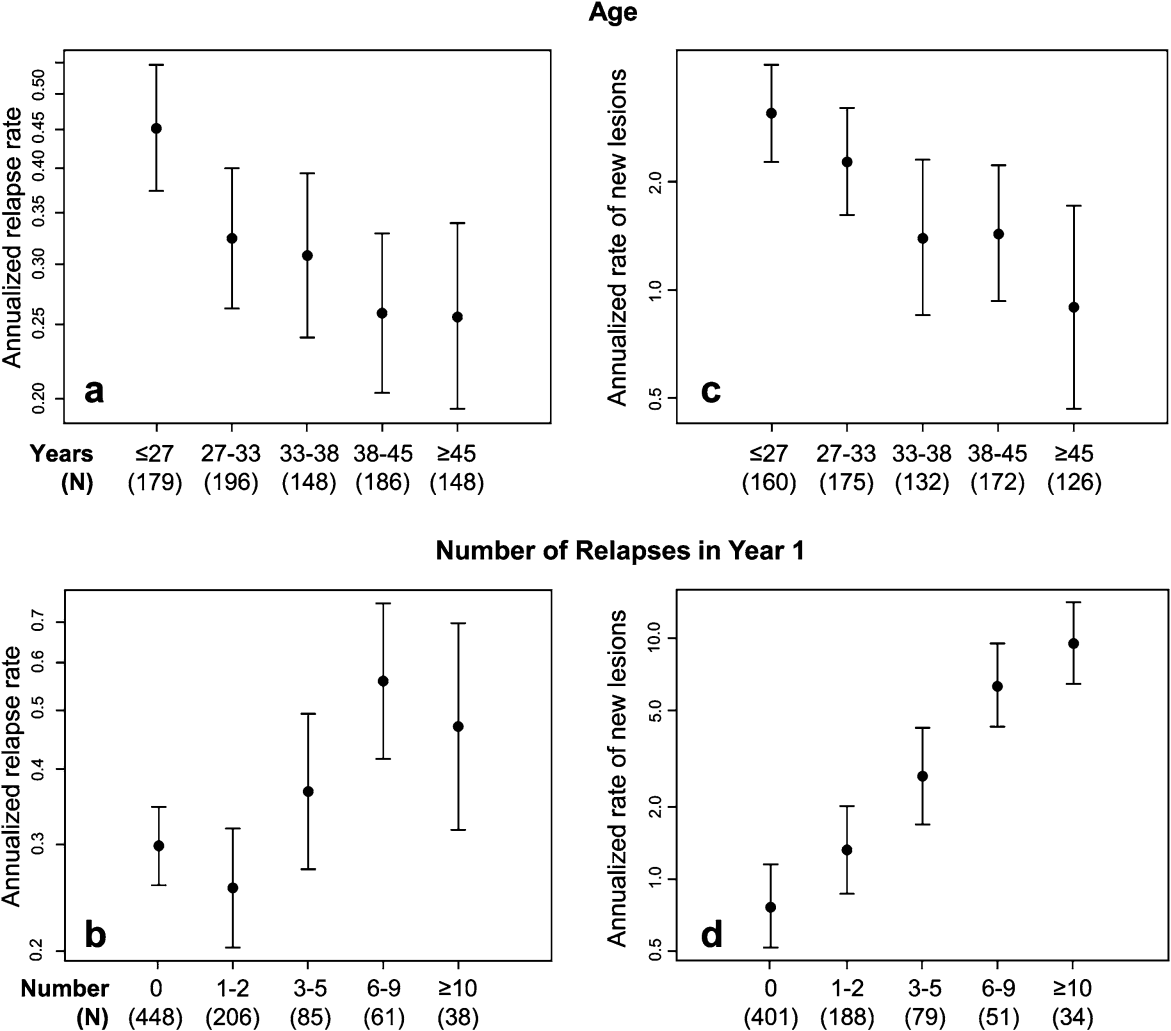
Impact of age and number of new lesions in year 1 on post-year 1 relapse and new lesion rates. Relationship between categorized age and number of new lesions in year 1 that significantly predicted post-year 1 relapse rates (**a**, **b**) and rates of new lesions (**c**, **d**) in both univariate and multivariate models

For the analysis of relapse data, there were 857 patients who had a mean time on study of 2.4 years. As shown in Table 1, for the univariate analysis, the baseline variables of a younger age (Fig. 1a), the presence of any Gd+ lesions, a higher number of relapses in the 2 years prior to trial enrollment, a higher T2 lesion volume, and a higher black hole lesion volume were each associated with a higher post-year 1 relapse rate. After 1 year of treatment, the variables of a higher number of new lesions on MRI and the occurrence of relapses predicted a higher post-year 1 relapse rate (Fig. 1b). By contrast, higher NAb titers were not significantly associated with the post-year 1 relapse rate and, in fact, those trends which did exist were for higher NAb titers to be associated with lower relapse rates (Table 1). EDSS progression was not significantly associated although the trend was for EDSS progression to be associated with a higher post-year 1 relapse rate (Table 1). In the multivariate analysis, the only variables that remained as significant independent predictors of a higher relapse rate in the final regression equation were younger age at baseline, higher number of relapses in the 2 years prior to trial onset, higher number of new MRI lesions in year 1, and the occurrence of relapses in year 1. Nevertheless, the contribution to the final regression equation by relapses prior to the randomized trial was only marginal and much reduced from its apparent contribution in the univariate analysis (Table 1).

**Table 1:** Predictors of post-year 1 relapse rate and MRI activity (estimate [95 % CI], *p* value)

		Relapse rate (*N* = 857)		MRI activity (*N* = 765)	
		Univariate^a^	Multivariate^b^	Univariate^a^	Multivariate^b^
Predictors at baseline before treatment					
Age		**−0.02 [−0.03, −0.01],** ***p*** **=** **0.002**	**−0.02 [−0.03, 0.00],** ***p*** **=** **0.011**	**−0.04 [−0.06, −0.02],** ***p*** **<** **0.0001**	**−0.04 [−0.05, −0.02],** ***p*** **<** **0.0001**
Male sex		−0.17 [−0.44, 0.10], *p* = 0.21	–	0.20 [−0.18, 0.59], *p* = 0.30	–
Presence of Gd+ lesions		**0.26 [0.02, 0.50],** ***p*** **=** **0.032**	–	**0.83 [0.45, 1.21,],** ***p*** **<** **0.0001**	–
Baseline EDSS		0.09 [−0.00, 0.19], *p* = 0.061	–	0.00 [−0.15, 0.15], *p* = 0.998	–
Number of relapses in past 2 years		**0.25 [0.14, 0.37],** ***p*** **<** **0.0001**	**0.13 [0.02, 0.24],** ***p*** **=** **0.024**	0.16 [−0.03, 0.35], *p* = 0.10	–
Disease duration		−0.01 [−0.03, 0.01], *p* = 0.56	–	−0.03 [−0.07, 0.01], *p* = 0.12	–
Baseline T2 lesion volume		**0.01 [0.00, 0.02],** ***p*** **=** **0.005**	–	**0.02 [0.01, 0.03],** ***p*** **=** **0.001**	**0.01 [0.00, 0.02],** ***p*** **=** **0.011**
Black hole lesion volume		**0.03 [0.01, 0.06],** ***p*** **=** **0.017**	–	0.03 [−0.01, 0.07], *p* = 0.18	–
Predictors within 1 year of treatment					
New MRI lesions in year 1	1–2	−0.16 [−0.48, 0.16], *p* = 0.33	−0.12 [−0.41, 0.17], *p* = 0.43	**0.54 [0.07, 1.00],** ***p*** **=** **0.025**	0.37 [−0.08, 0.82], *p* = 0.11
	≥3	**0.41 [0.13, 0.69],** ***p*** **=** **0.004**	**0.29 [0.02, 0.55],** ***p*** **=** **0.034**	**1.89 [1.52, 2.26],** ***p*** **<** **0.0001**	**1.51 [1.13, 1.88],** ***p*** **<** **0.0001**
NAb titer at year 1	20–400	−0.06 [−0.34, 0.22], *p* = 0.67	–	**0.90 [0.51, 1.28],** ***p*** **<** **0.0001**	**0.71 [0.40, 1.03],** ***p*** **<** **0.0001**
	>400	−0.24 [−0.86, 0.38], *p* = 0.45	–	**1.00 [0.36, 1.65],** ***p*** **=** **0.003**	**0.97 [0.45, 1.50],** ***p*** **=** **0.0003**
Confirmed EDSS progression in year 1		0.27 [−0.17, 0.72], *p* = 0.23	–	−0.63 [−1.67, 0.41], *p* = 0.23	–
Relapses in year 1		**1.17 [0.94, 1.39],** ***p*** **<** **0.0001**	**1.03 [0.80, 1.26],** ***p*** **<** **0.0001**	0.30 [−0.09, 0.68], *p* = 0.13	–

Numbers represent regression coefficients. Relapse rate and MRI activity are the dependent variables in the regression equations. Coefficients greater than 0 indicate a positive association between the predictor and the dependent variable

*95* *% CI* 95 % confidence interval [shown in brackets], *EDSS* expanded disability status scale, *Gd*+ gadolinium enhancing, *dashes* indicate variables not selected by the stepwise multivariate procedure

^a^ *p* values not corrected for multiple comparisons, values that crossed the threshold for significance are in bold

^b^ Only predictors with *p* < 0.05 were included

765 patients were available for the analysis of the MRI data. As demonstrated in Table 1, for the univariate analysis, the baseline variables of younger age (Fig. 1c), the presence of any baseline Gd+ lesions, and higher baseline T2 lesion volume were each associated with a higher post-year 1 relapse rate. After 1 year of treatment, the variables of higher number of new MRI lesions in year 1 (Fig. 1d) and higher NAb titer at year 1 predicted a higher MRI activity rate. EDSS progression and relapses in the first year were not significantly associated although the trends were for higher relapses and non-occurrence of EDSS progression to be associated with higher MRI activity. In the multivariate analysis, the only variables that remained as significant independent predictors of a higher MRI activity rate in the final regression equation were younger age at baseline, higher baseline T2 volume, higher number of new MRI lesions in year 1, and higher NAb titer at year 1.

Interestingly, it seems that relapses during therapy were better predictors of future relapses than they were of future MRI activity (Table 1). Similarly, MRI activity seemed to have been a better predictor of future MRI activity than it was of future relapses (Table 1).

## Discussion

The introduction of new drugs as MS therapeutics has been accompanied by the prospect of both improved efficacy and increased toxicity [6]. In such a circumstance, physicians require guidance about the optimal time to switch a patient from a first-line therapy to a new therapy that is potentially more effective but also potentially more risky. There is some evidence that brain MRI could be used to indicate treatment response for IFNβ formulations [7]; however, this issue is highly controversial. To help address this question, we undertook a subgroup analysis of the BEYOND study of those patients treated with the standard dose of IFNβ-1b to search for factors that may be indicative of an insufficient response to treatment. Because IFNβ-1b treatment unequivocally reduces (on average) both clinical and MRI evidence of disease activity [2, 8], the identification of those factors (while on therapy), which predict a poor post-treatment outcome, might be very helpful to clinicians in identifying those patients who are in need of more aggressive management.

In the present study, several clinical and brain MRI predictors (both at baseline and during treatment) were identified by univariate analysis as being associated with higher levels of disease activity during the subsequent period of observation. By contrast, the number of variables was greatly reduced when we used a multivariate approach to look for independent contributors. Thus, both the relapse activity before treatment and, to a much greater extent, the relapses during the first year of treatment were significantly associated with higher post-year 1 relapse rates. However, none of these predictors were selected in the multivariate model for prediction of MRI lesion rates. Similarly, both the baseline T2 hyperintense lesion volume and, especially, increasing MRI activity during the first year of therapy were significant independent predictors of future MRI activity. However, although the highest category of MRI activity (≥3 new lesions) was weakly predictive of future relapses, none of the other MRI variables were significantly associated with future relapses. Also, the presence of NAbs during the first year of therapy was a significant predictor of future MRI activity. Nevertheless, consistent with previously published observations from BEYOND and from other studies [9, 10], there was not even a hint of an impact of NAbs on future relapse activity (Table 1).

These findings are similar to, but distinct from, those reported previously [11–13]. In a study of 222 patients, the authors reported that the combination of relapses together with the finding of new active MRI lesions was particularly important prognostically [12]. In the present study, also, both relapses and MRI activity were important for prognosis but seemed to predict different outcomes (i.e., relapses predicted future relapses and MRI predicted future MRI activity). This kind of disconnect between the clinical state and MRI has been noted previously and has been referred to as the clinico-radiological paradox [14]. It is also similar to the disconnect, which has been observed with respect to the correlation between the short-term outcome measures and long-term disability [15]. Thus, in the 16-year follow-up of the pivotal IFNβ-1b trial, the only significant on-study predictors of disability outcomes were the clinical measures of attack rate and short-term disability [15]. By contrast, all of the on-study MRI variables were not correlated with outcome [15].

In this context, because in the present study the occurrence of relapses on therapy was strongly predictive of future relapses, and because relapses seem to be predictive of future disability [16], the results of the present study suggest that clinicians, faced with a patient who continues to experience clinical activity, should seriously consider an alteration in their therapeutic approach to that patient. MRI activity might contribute to this decision only if the activity is substantial. Nevertheless, at the moment, such a conclusion must be considered only tentative. The observation period of the BEYOND study was short and the disease activity (observed in this study) was quite low. Both of these factors limit the generalizability of these observations to long-term disability progression. Moreover, the relationship of clinical relapses and/or MRI activity to long-term disability progression is controversial and requires further study [4, 17, 18].
